# Properties of Doublecortin Expressing Neurons in the Adult Mouse Dentate Gyrus

**DOI:** 10.1371/journal.pone.0041029

**Published:** 2012-09-05

**Authors:** Jay Spampanato, Robert K. Sullivan, Fabrice R. Turpin, Perry F. Bartlett, Pankaj Sah

**Affiliations:** The Queensland Brain Institute, The University of Queensland, Brisbane, Australia; Institut National de la Santé et de la Recherche Médicale, France

## Abstract

The dentate gyrus is a neurogenic zone where neurons continue to be born throughout life, mature and integrate into the local circuitry. In adults, this generation of new neurons is thought to contribute to learning and memory formation. As newborn neurons mature, they undergo a developmental sequence in which different stages of development are marked by expression of different proteins. Doublecortin (DCX) is an early marker that is expressed in immature granule cells that are beginning migration and dendritic growth but is turned off before neurons reach maturity. In the present study, we use a mouse strain in which enhanced green fluorescent protein (EGFP) is expressed under the control of the DCX promoter. We show that these neurons have high input resistances and some cells can discharge trains of action potentials. In mature granule cells, action potentials are followed by a slow afterhyperpolarization that is absent in EGFP-positive neurons. EGFP-positive neurons had a lower spine density than mature neurons and stimulation of either the medial or lateral perforant pathway activated dual component glutamatergic synapses that had both AMPA and NMDA receptors. NMDA receptors present at these synapses had slow kinetics and were blocked by ifenprodil, indicative of high GluN2B subunit content. These results show that EGFP-positive neurons in the DCX-EGFP mice are functionally immature both in their firing properties and excitatory synapses.

## Introduction

The mammalian hippocampal dentate gyrus is part of the limbic system and plays an essential role in learning and memory formation. In contrast to other brain areas, this region has a delayed development and, in rodents, the normal production of granule cells peaks about seven days postnatal and continues through the first postnatal month [1]. However, unlike in most other brain regions, the generation and development of new neurons in the dentate gyrus continues beyond the normal developmental period persisting at a slower rate throughout adult life in both rodents and humans [2], [3]. This neurogenesis occurs in the sub granular zone (SGZ) between the granule cell layer and the hilus [4]. In mature animals, these newly generated neurons integrate into the existing circuitry and are thought to play important roles in learning and memory formation as well as repair [5].

Electrophysiological recordings and morphological reconstruction of granule cells in the dentate gyrus from immature rodents have demonstrated that these neurons vary from small size, high input resistance cells with small dendritic trees that do not fire action potentials, to mature neurons with large dendritic trees that fire trains of action potentials. This profile is thought to reflect cells at different stages of a stereotypical developmental program from early postmitotic stages to mature neurons [6], . In adult animals, recordings from neurons in the granule cell layer of the dentate gyrus have revealed cells with a similar distribution of properties to that seen in immature animals leading to the suggestion that these represent newly generated neurons at different stages of maturation in the adult [9], [10], [11], [12], [13]. However, as the rate of neurogenesis is low in adult animals [14], cells thought to be immature neurons are encountered relatively infrequently. This makes characterizing these cells in adult animals difficult, and their developmental status is only apparent once recording is complete. It is therefore very useful to be able to identify newborn neurons directly, prior to experimentation.

To visually identify newborn neurons in the adult, two different approaches have been used. In the first, retroviral-mediated expression of enhanced green fluorescent protein (EGFP) has been used to label the newborn granule cells 13,15,16,17,18. In the second approach, transgenic mouse lines have been found that express EGFP in newborn granule cells [19], [20], [21], [22], [23]. These studies have suggested a timeline of electrophysiological, immunohistochemical and anatomical phases of development from the mitotic progenitor stage to differentiated immature granule cells and finally fully mature granule cells [21], [24], [25]. However, studies based on neurons marked using retroviral transfection are complicated by the transfection procedure that requires injection of viral particles using neurosurgical techniques. Retroviral transfection is effective in identifying adult newborn neurons but, as pathological, pharmacological and environmental manipulations all modulate adult neurogenesis [26], [27], the relationship of newborn neurons found following viral transfection to those generated normally is not clear. Studies using transgenic animals where EGFP is used to identify newborn neurons eliminates this problem, however, in the lines studied to date, expression of EGFP in newborn neurons has been somewhat serendipitous in that while immature cells clearly express EGFP in these animals, the role of the particular promoters in neural development is not clear [19], [23]. That is, the targeted neurons remain EGFP negative while the newborn granule cells are EGFP-positive. Notably, as this is an area of active and ongoing research, new more powerful fate-mapping transgenic tools are continually being developed [28], [29].

During neurogenesis, newborn cells progress though several stages of development before attaining their final mature phenotype. These stages can be differentiated by the temporal expression pattern of different sets of proteins [4]: radial glia-like progenitors express glial fibrillary acidic protein (GFAP), whereas immature granule cells that are beginning migration, dendritic growth and targeting express the proteins doublecortin (DCX), poly-sialated neural cell adhesion molecule (PSA-NCAM) and calretinin [25]. Finally, when mature, dentate granule cells turn off DCX and PSA-NCAM and express calbindin in place of calretinin [25]. As DCX is only present in the early stages of differentiation, expression of fluorescent markers using the DCX promoter presents the possibility of examining the properties of newly generated neurons in the adult dentate gyrus. In the present report we characterize the properties of newborn neurons in the adult dentate gyrus of a DCX-EGFP mouse line developed by the Gene Expression Nervous System Atlas BAC transgenic project (GENSAT) [30]. These mice were created in the outbred Swiss Webster strain that has some distinct advantages over inbred strains, being better suited for large drug discovery studies that require a genetically heterogeneous population. We show that EGFP-expressing granule cells in these animals are newborn neurons and describe their electrophysiological, immunohistochemical and anatomical properties.

## Results

**Figure 1:**
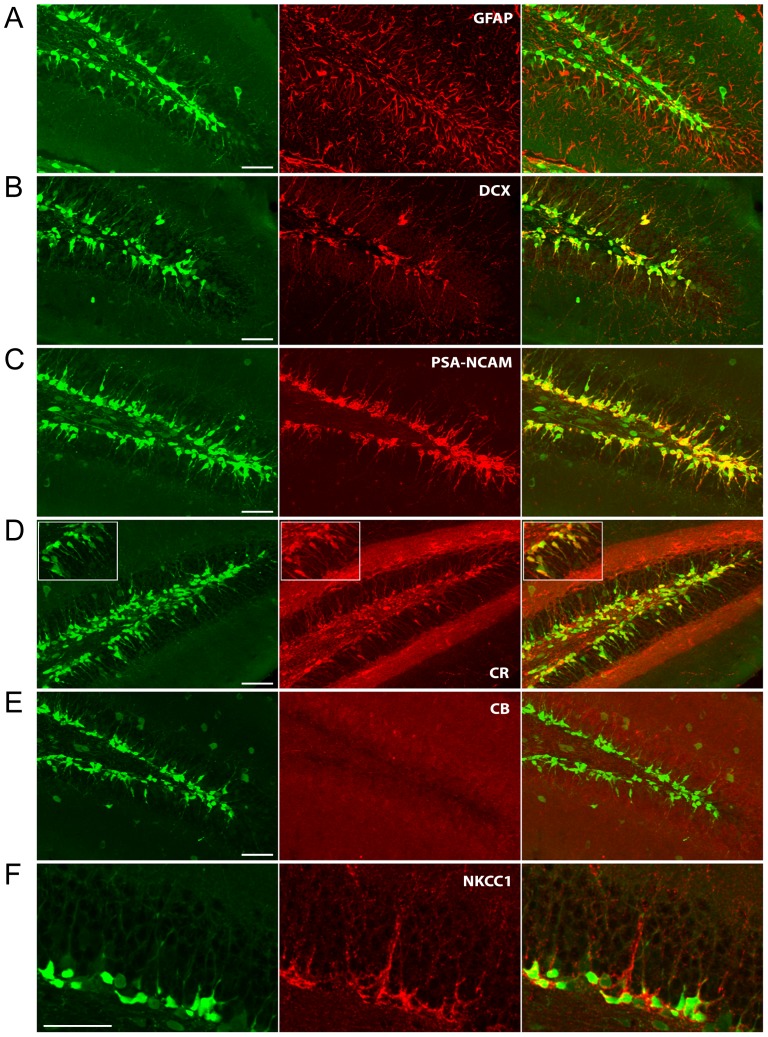
EGFP-expressing Granule Cells Are Newborn Neurons. Coronal sections through the dentate gyrus were labeled with antibodies for EGFP (left panels, green channel) and proteins known to be expressed at various time points of dentate granule cell development (center panels, red channel). Each image represents a maximum intensity compressed Z-stack. A. There was no colocalization of EGFP with the Glial Fibrillary Acidic Protein (GFAP), confirming that the EGFP-expressing cells are postmitotic. B. & C. Both the dendritic growth markers Doublecortin (DCX) and Poly-Sialated Neural Cell Adhesion Molecule (PSA-NCAM) colocalize with EGFP expression (merged right panels, yellow). D. & E. Calretinin (CR), the calcium binding protein expressed by immature granule cells, is also present in EGFP-expressing granule cells (see inset), while Calbindin (CB), expressed in mature granule cells, is not detectable. F. The sodium-potassium-chloride cotransporter NKCC1, known to be important for the proper development of immature granule cells, is also detectable in association with EGFP-expressing cells. In each case: objective = 20×, scale bar = 50 µM.

**Figure 2:**
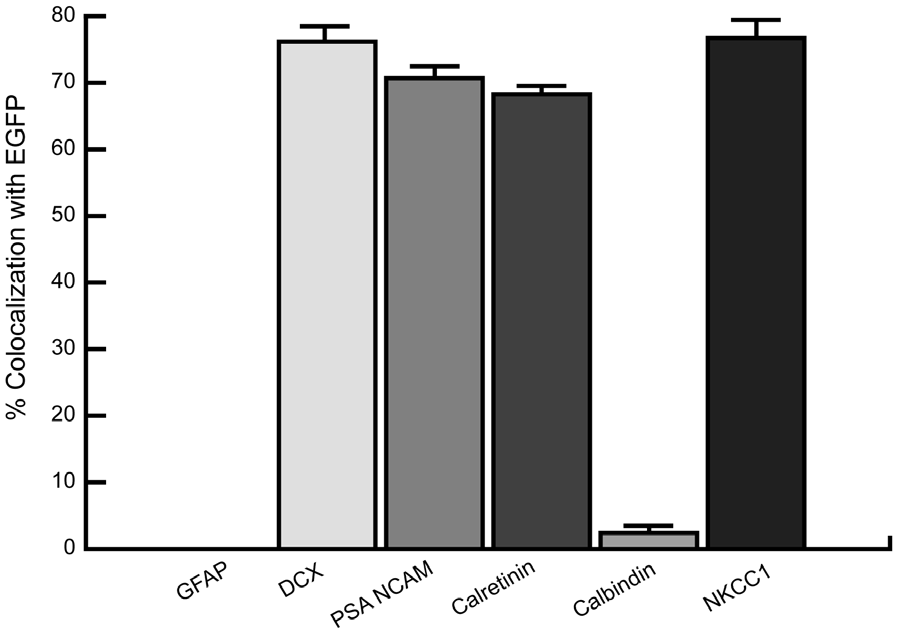
Quantification of co-expression of EGFP with markers for newborn neurons in the dentate gyrus demonstrates significant colocalization between EGFP and Doublecortin (DCX), Poly-Sialated Neural Cell Adhesion Molecule (PSA-NCAM), Calretinin (CR), and Sodium-potassium-chloride cotransporter NKCC1 but not Glial Fibrillary Acidic Protein (GFAP) or Calbindin (CB).

We first investigated the neurogenic status of EGFP-expressing dentate gyrus granule cells in the DCX-EGFP mouse line. As different developmental stages can be differentiated by the expression of unique sets of proteins [4], we first verified the status of the EGFP-expressing neurons by testing for expression of DCX, GFAP, PSA-NCAM, calretinin and calbindin (Fig. 1). Consistent with the fact that DCX expression marks cells that develop into neurons, we found no overlap between GFAP and EGFP expression (Fig. 1A). Instead, EGFP-positive granule cells expressed the dendritic growth markers DCX and PSA-NCAM (Fig. 1B, C), as well as the immature dentate gyrus granule cell marker calretinin (Fig. 1D). Consistent with this immature phenotype, there was no overlap between EGFP and calbindin expression (Fig. 1E). From this immunohistochemical profile we conclude that in this mouse strain, EGFP-expressing cells are indeed newborn granule cells in the post-differentiation stage of development. Newborn granule cells in the adult dentate gyrus have also been found to require a high intracellular chloride concentration that is maintained by the sodium-potassium-chloride cotransporter (NKCC1) [31]. Further confirming their early stage of development, EGFP-positive cells were also found to express NKCC1 (Fig. 1F). Using unbiased stereological analysis (Fig. 2) we found that EGFP co-localized with DCX, PSA-NCAM, calretinin and NKCC1 in 78%, 71%, 68%, and 77% of cells respectively. Expression of calbindin was found in only 2.7% of EGFP-positive cells and no EGFP was detected in glial cells labeled for GFAP. This protein expression profile is consistent with newborn and immature granule cells in their first three weeks of postmitotic differentiation [21], [25]. Consistent with this conclusion and in accord with previous finings [32], BrdU pulse labeling showed that two weeks following BrdU injection all cells labeled for BrdU coexpressed EGFP with the majority of cell bodies being present within the first third of the cell body layer and a few cell bodies being present in the middle third of the cell body layer (data not shown).

**Figure 3:**
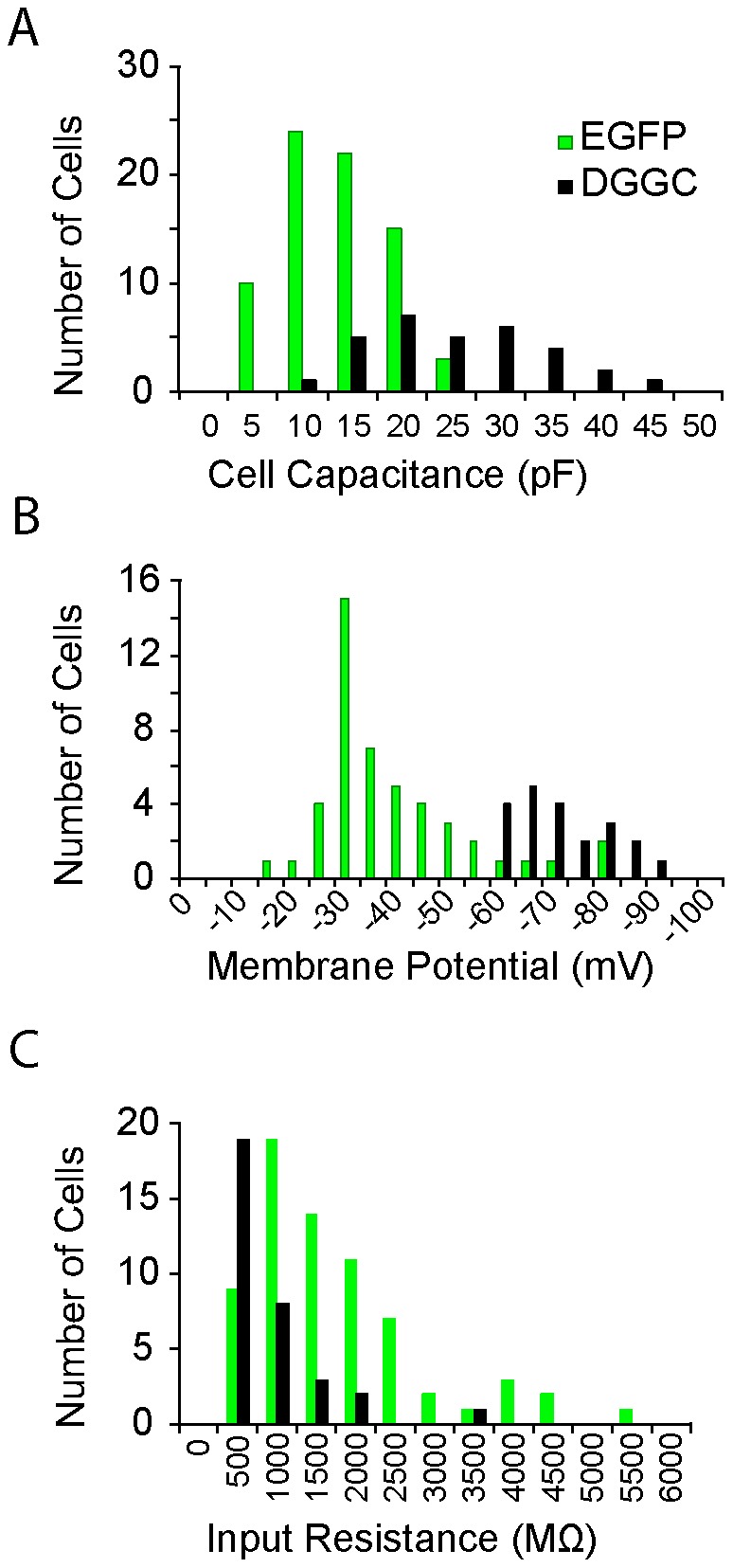
Intrinsic Properties of EGFP-expressing and Mature Granule Cells. Histograms of the whole-cell capacitance, resting membrane potential and input resistance demonstrate that EGFP-expressing granule cells (green lines) are typically smaller, more depolarized (though inaccurately measured, see text) and have a higher input resistance than mature dentate gyrus granule cells (DGGC, black lines).

Having established that the EGFP-positive granule cells in the adult are immature neurons, we next assessed their electrotonic properties, the synaptic inputs they receive, and compared them to mature dentate gyrus granule cells. Recording were obtained from 74 EGFP-positive and 31 EGFP-negative (presumed mature), dentate gyrus granule cells. In each case, EGFP-negative cells were selected from the outer 1/3 of the dentate granule cell layer, furthest from the neurogenic sub-granule cell zone and closest to the molecular layer. As expected, immature (EGFP-positive) granule cells were significantly smaller than mature granule cells with an average whole-cell capacitance of 11.2±5.3 pF compared to 23.2±9.0 pF respectively (p<0.001, Student's t-Test, table 1, Fig. 3A). EGFP-positive granule cells also appeared to have a more depolarized resting membrane potential (−40.4±14.8 mV) than mature granule cells (−73.6±8.8 mV, p<0.001, Student's t-Test, table 1, Fig. 3B). However, this apparent depolarized membrane potential is likely to be inaccurate as EGFP-positive granule cells displayed extremely high input resistances with a mean of 1.52±1.10 GΩ compared to mature granule cells that had an input resistance of 0.27±0.24 GΩ (p<0.001, Student's t-test, table 1, Fig. 3C). This high input resistance will introduce an error in measurement of the membrane potential due to the shunt introduced by the leak conductance at the pipette seal [33], [34].

**Table 1:** Comparison of electrotonic properties of EGFP-positive granule cells and mature dentate gyrus granule cells.

Mature dentate gyrus neurons			
	WCC (pF)	Vm (mV)	Ri (GΩ)
Mean	23.2	−73.6	0.27
SD	9	8.8	0.24
n	25	21	26

EGFP-positive neurons			
Mean	11.2	−40.4	1.52
SD	5.3	14.8	1.1
n	55	48	52
t-Test	1.30E-06	1.70E-16	2.00E-12

**Figure 4:**
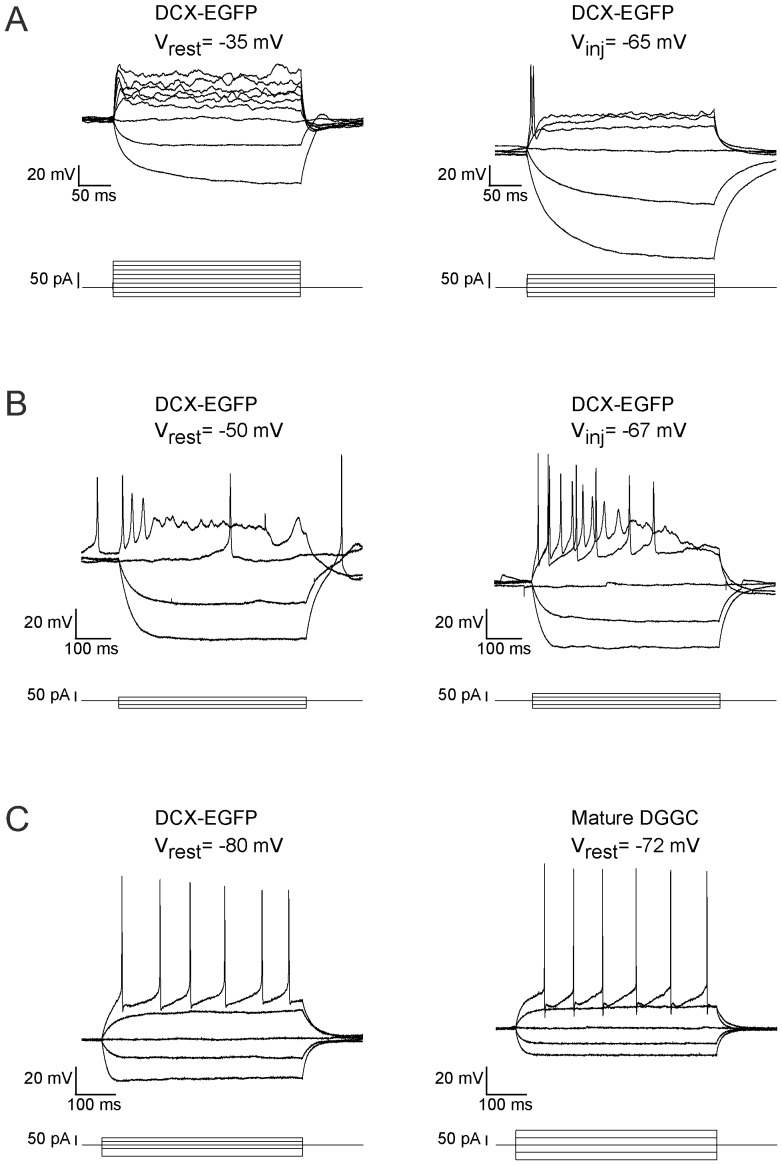
Firing Properties of EGFP-expressing Granule Cells. The firing properties of EGFP-expressing granule cells in the DCX-EGFP mouse indicate that the cells are at various stages of electrophysiological maturation. A. The majority of recorded cells had an apparent (though inaccurately measured, see text) depolarized resting membrane and failed to fire an AP (left) during successive depolarizing square-pulse current injections (corresponding current injections are indicated below each set of voltage traces). However, when a hyperpolarizing current was used to artificially maintain a more negative resting membrane potential some cells were capable of firing a single AP (right). B. Some EGFP-expressing granule cells were capable of firing more than one AP from rest (left) and could be converted to firing multiple APs when artificially maintained at a more negative resting potential (right). C. A few EGFP-expressing granule cells fired sustained trains of APs (left) like mature granule cells (right). These cells were often dim in comparison to the first group, and located a couple of cell layers from the SGZ towards the molecular layer. Mature granule cells were recorded at the outer lip of the granule cell layer adjacent to the molecular layer. In each panel (A–C) both sets of traces (left and right) are plotted on the same y-axis for that panel.

EGFP-positive granule cells could be divided in to three categories based on their ability to fire action potentials. The large majority (31/40) fired either no action potentials or a mini spikelet in response to depolarizing current injections (Fig. 4A, left). Some of these cells (9/31) could be converted to fire a single action potential when they were hyperpolarized by current injection (Fig. 4A, right). This phenotype is consistent with that described for some neurons found at the border of the granule cell layer suggesting they are early postmitotic dentate gyrus granule cells [9], [21]. The remaining EGFP-expressing granule cells (9/40) were capable of firing a few action potentials from rest. These cells sometimes also fired a rebound action potential immediately following the first hyperpolarizing current injection (Fig. 4B, left). Hyperpolarizing these cells with current injection could convert some of these cells to fire longer trains of action potentials, though most were still unable to maintain a sustained train of APs (Fig. 4B, right). This firing pattern is more consistent with immature granule cells in at least their second or third week of development [21]. To the naked-eye, prior to obtaining a whole-cell patch clamp configuration, these cells appeared less bright than the first group; likely a result of the cessation of EGFP expression as DCX expression is turned off in the later stage of development. These cells also appeared to have migrated further into the dentate granule cell layer. Finally, a few of these EGFP-expressing granule cells were capable of firing a sustained train of action potentials similar to that observed for mature dentate gyrus granule cells (Fig. 4C).

**Figure 5:**
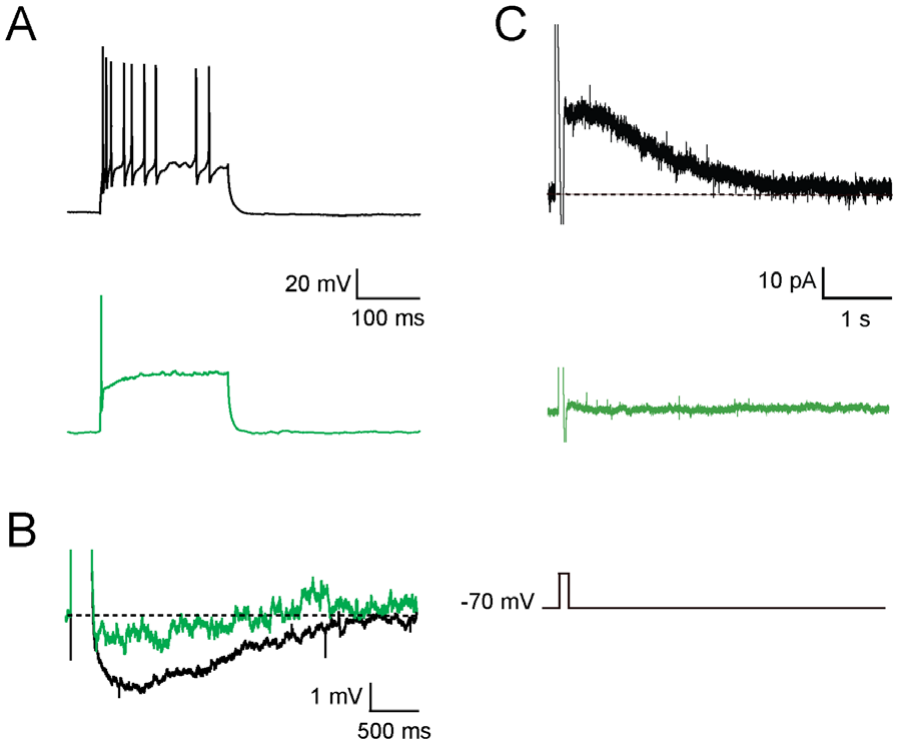
EGFP-expressing neurons do not show a slow afterhyperpolarization. A. A train of action potentials fired by a mature granule cell (black, top) demonstrating spike-frequency adaptation. Most immature EGFP-expressing granule cells do not fire trains of action potentials in response to suprathreshold current injections. B. Voltage traces elicited in response to a large current injection for EGFP-expressing dentate gyrus granule cells (green) and a mature dentate gyrus granule cell (black), demonstrating the slow AHP is not present in immature granule cells. C. The slow AHP can also be seen in the outward current trace in response to a voltage step to 0 mV (bottom trace) for mature granules cells (black, top) but is absent in the immature cells (green, middle).

**Figure 6:**
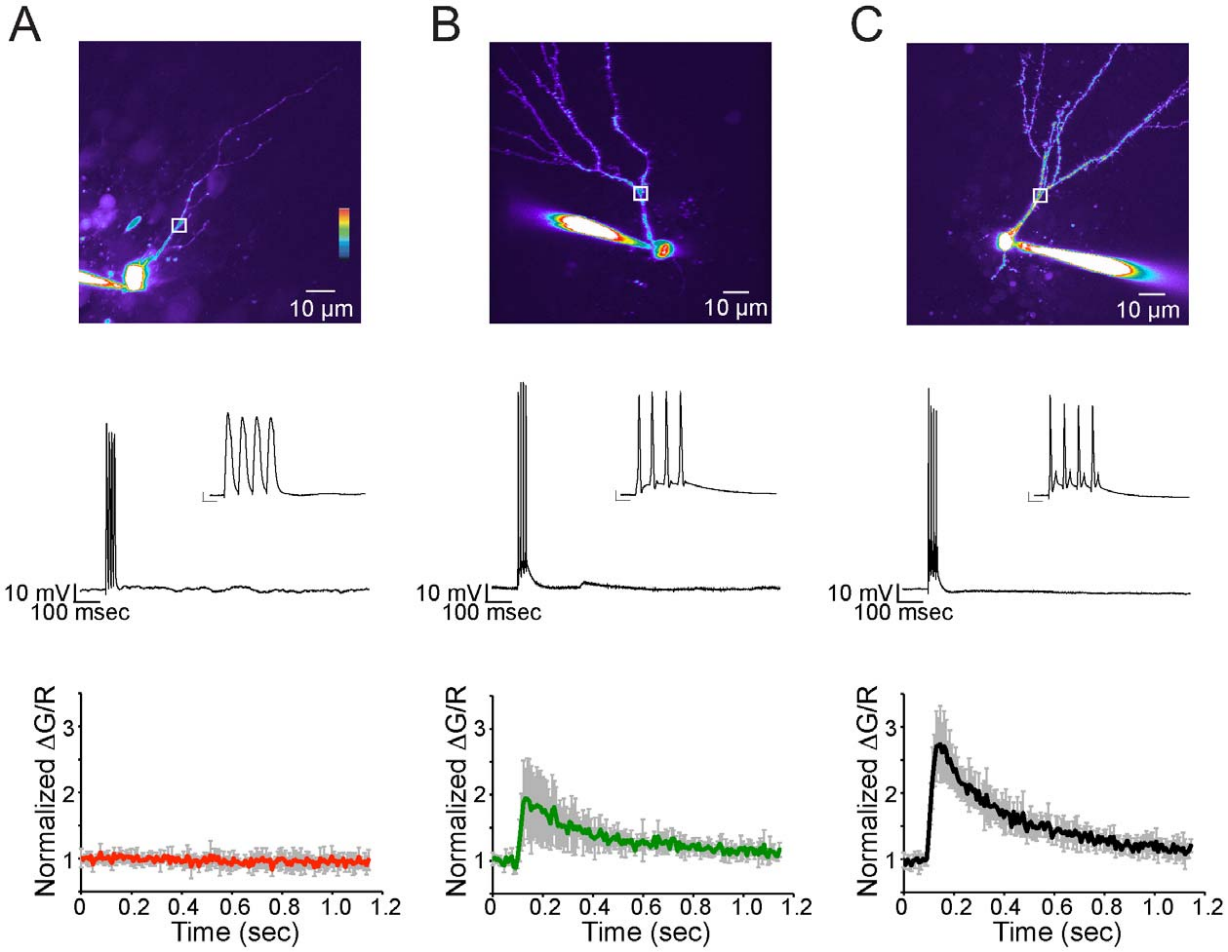
Dendritic Calcium Signaling in EGFP-expressing Granule Cells. An intracellular calcium rise was elicited by a train of 4 APs at 100 Hz and measured at approximately 20–30 µM from the soma, before the first branch point of the main apical dendrite (white boxes). A. One group of immature EGFP-expressing granule cells, that were unable to fire APs (middle) had no detectable dendritic calcium rise (bottom, average and standard deviation, n = 5) in response to current injections that elicited a change in membrane potential similar in magnitude to an AP. B. More mature looking EGFP-expressing granule cells with sparsely spiny dendrites (top) were able to fire 4 APs (middle) that resulted in a detectable rise in intracellular calcium concentration in the apical dendrite (bottom, average and standard deviation, n = 5). C. Mature granule cells were densely spiny and 4 APs elicited a relatively large rise in dendritic calcium (bottom, average and standard deviation, n = 6). (Inset scale bars are 10 mV and 10 ms).

As with many other central neurons [35], some adult dentate gyrus granule cells respond to current injection by discharging trains of action potentials that show spike frequency adaptation (Fig. 5A) that is largely mediated by activation of calcium-dependent potassium currents and the resultant slow afterhyperpolarization (AHP) [36]. Newborn neurons that fired action potentials did not express a slow AHP (Fig. 5B). In mature granule cells, the slow AHP, triggered by a single square pulse current injection, measured at −70 mV, had an amplitude of 2.0±0.4 mV (n = 8) compared to 0.86±0.23 mV (n = 6) in newborn neurons (p<0.03, Student's t-test). In this recording configuration these newborn neurons fired only a single AP. However the lack of the slow AHP is not the result of evoking fewer action potentials in these neurons as, under voltage clamp, a step to 0 mV for 100 ms also failed to evoke an outward current while evoking a clear slow I_AHP_ current [35] in mature dentate granule cells (Fig. 5C). Moreover, this lack of a slow AHP is not due to the absence of calcium channels as cells that fired action potentials displayed robust calcium transients in the dendritic tree (Fig. 6). The slow AHP in these newborn cells that were triggered to fire 4 APs by rapid, successive supratheshold current injections had an amplitude of 0.28±0.19 mV (n = 5) compared to 1.74±1.1 mV (n = 5) for mature granule cells triggered in the same manner. When the two data sets are combined, the slow AHP in newborn neurons had an amplitude of 0.60±0.50 mV (n = 11) compared to 1.93±0.99 mV (n = 13) for mature granule cells (p<0.001, Student's t-test).

We next examined the calcium-buffering properties of newborn neurons. Neurons were loaded with the high affinity calcium sensitive dye: Oregon green BAPTA-1 (OGB-1) and the calcium response measured in response to a single action potential or a train of action potentials. Granule cells characterized as immature neurons based on their electrophysiological properties have previously been shown to have a different calcium buffering capacity in response to back propagating action potentials compared to mature dentate gyrus granule cells [12]. We therefore investigated action potential back propagation in newborn and immature granule cells in the DCX-EGFP mouse. Action potential back propagation was measured as a rise in intracellular calcium concentration 20–30 µM from the soma along the apical dendrite, just before the first dendritic branch point. A burst of 4 action potentials was elicited at 100 Hz by brief suprathreshold current injections, and changes in calcium concentration were measured as an increase in green fluorescence (OGB-1) as compared to the calcium insensitive red fluorescent dye Alexa-594. The group of immature granule cells that were unable to fire an action potential from rest was observed to have no rise in intracellular calcium concentration in response to large depolarizing (voltage response>0 mV) current injections (Fig. 6A, n = 5). Though the cells did not fire action potentials, the current injections elicited a change in membrane voltage similar in magnitude to that of an action potential (Fig. 6A, center). Immature granule cells that did fire action potentials in response to current injections also exhibited a summated rise in intracellular calcium concentration due to dendritic back propagation (Fig. 6B, n = 5).

The average sustained calcium rise for the duration of action potential firing (∼40 ms) in these newborn cells (normalized ΔG/R = 1.87±0.55, n = 5) was significantly smaller when compared to mature neurons (normalized ΔG/R = 2.69±0.48, n = 6, p<0.05, Student's t-test). However, the kinetics of the calcium transient decay were not significantly different between newborn (τ = 449±104 ms) and mature granule cells (τ = 432±99 ms).

**Figure 7:**
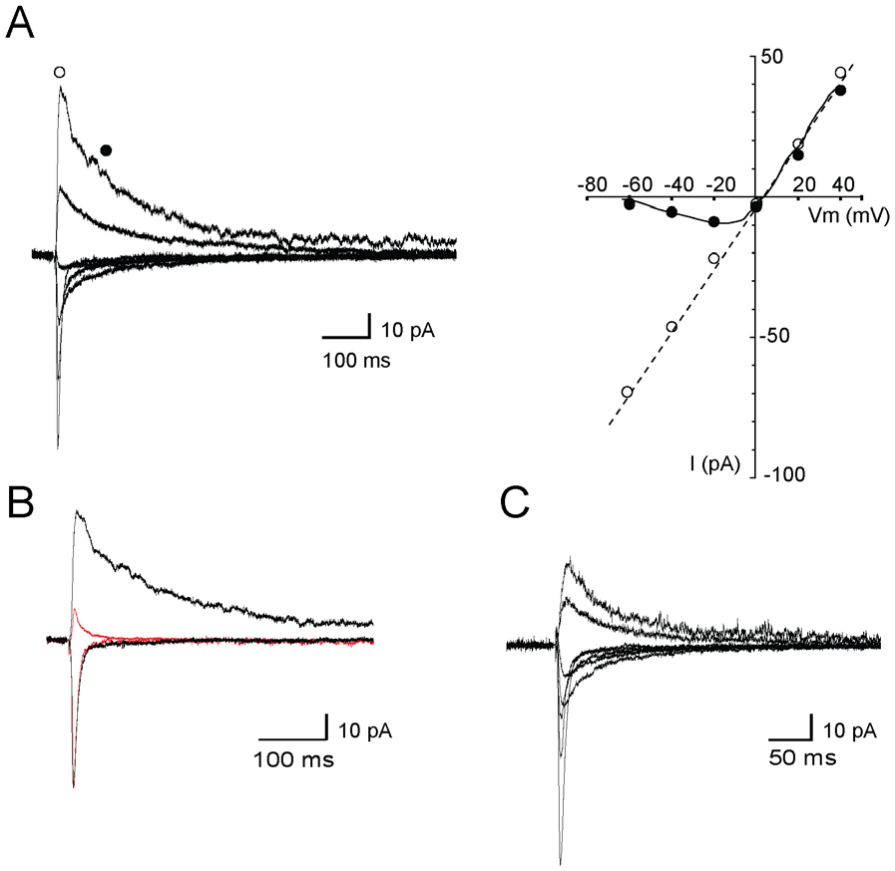
Dual Component Glutamatergic Synapses Onto Immature Granule Cells. A. Synaptic currents recorded in a EGFP-expressing dentate gyrus granule cell held at various membrane potentials between −60 mV and +40 mV, in response to medial perforant path stimulation. The current-voltage relationship (right) is plotted for currents measured at the peak (open circle) and 100 ms later (closed circles). Newborn dentate gyrus granule cells have dual component synapses that exhibit a linear component (AMPA+NMDA) and a component that is blocked at negative potentials (NMDA). B. Current traces at −60 mV and +40 mV before (black) and after (red) block of NMDA channels by d-AP5. C. Dual component synapse recorded from a mature dentate gyrus granule cell.

When EGFP-positive dentate gyrus granule cells were imaged, dendrites could be observed extending in to the molecular layer. Neurolucida drawings of filled cells were used for quantitative analysis to measure total dendritic length and spine counts. Most of the dendrites were reasonably spiny (0.4903±0.058 spines/µm), except for the proximal segments close to the cell body. The spine counts were approximately half of that reported for mature dentate cells (1.160±0.108 spines/µm) [37] and in some segments as low as 0.195 spines/µm. The total numbers of spines per cell was 537±7.50 and the total dendritic length, 1186.35±156.95 µm. We next tested for the presence of excitatory synaptic currents onto these neurons. Dentate granule cells receive two different entorhinal inputs, those from the medial perforant path and those from the lateral perforant path. Under voltage-clamp, excitatory postsynaptic currents (EPSCs) could be evoked by stimulation of either perforant pathway. At negative membrane potentials, stimulation evoked a fast inward current that reversed near 0 mV. Depolarization revealed a voltage-dependent slow component (Fig. 7A) that was selectively blocked by the NMDA receptor antagonist d-AP5 (30 µM; Fig. 7B). Thus, as in mature neurons (Fig. 7C) perforant path inputs also formed dual component glutamatergic synapses on newborn neurons.

**Figure 8:**
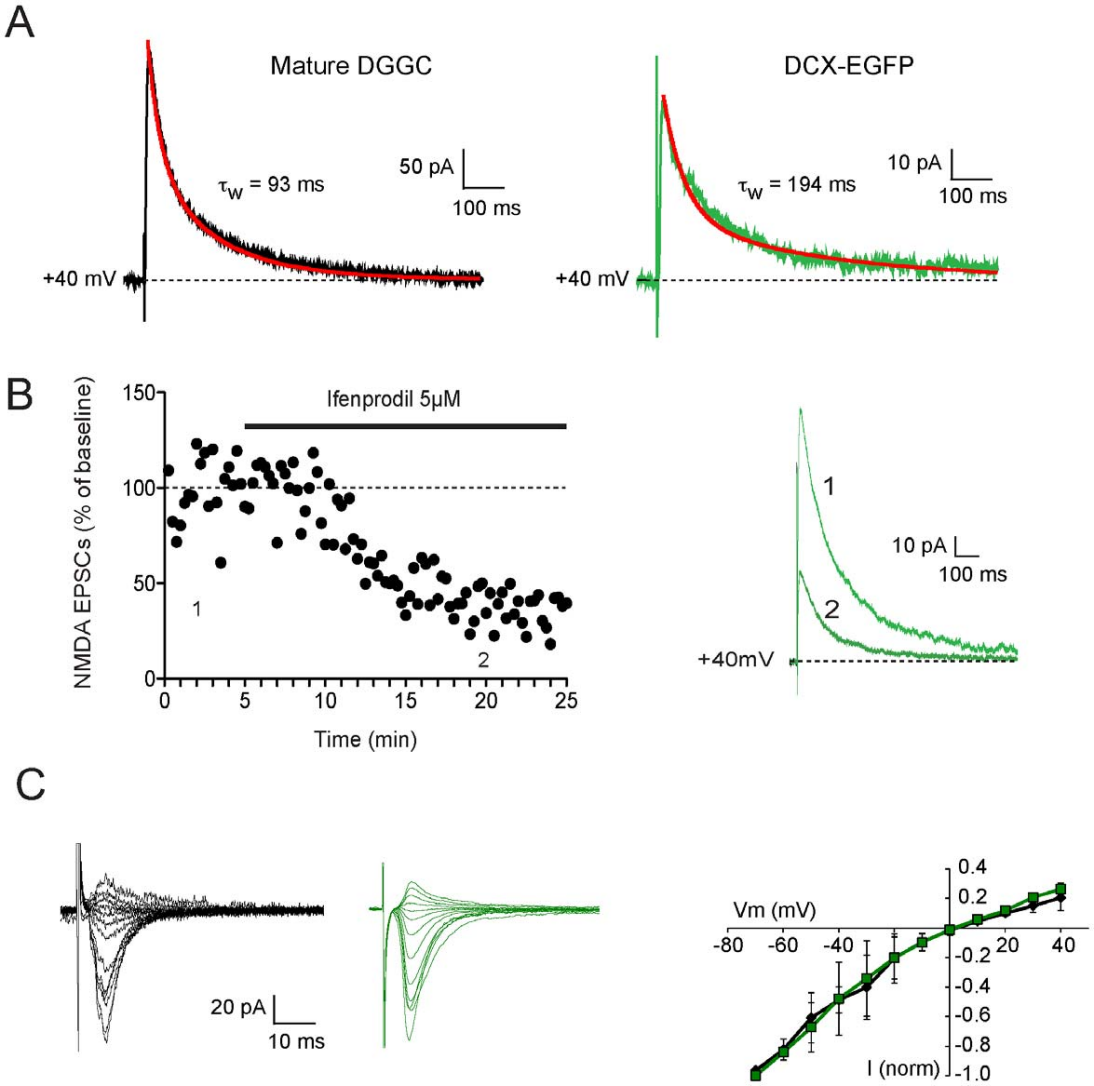
Both Immature and Mature Granule Cells Have Slow NMDA Kinetics and Rectifying AMPA Receptors in Swiss Webster Mice. A. NMDA currents recorded at +40 mV in response to medial perforant path stimulation in a mature granule cell (left) and from and EGFP-positive newborn neurons (right). Recordings were fit with a double exponential equation and the fit is overlaid (red lines). B. The NMDA receptor mediated EPSC in newborn neurons is blocked by ifenprodil. Panel on left shows the normalized NMDA-receptor mediated EPSC amplitude for one cell recorded at +40 mV before and following application of 5 µM ifenprodil. The EPSCs recorded at the indicated time points are superimposed on the right. C. AMPA-receptor mediated synaptic currents recorded over a range of membrane potentials from −80 to +40 mV following blockade of NMDA receptor currents with d-AP5. Currents were normalized to the peak current recorded at −80 mV and plotted against voltage to demonstrate the slight rectification at depolarized potentials in both mature (black traces) and newborn (green traces) granule cells. The peak current-voltage relationship for the two cell types are shown on the right.

NMDA receptors are tetramers that contain two obligatory GluN1 subunits and two of four GluN2 subunits (GluN2A-GluN2D). The GluN2 subunits confer distinct pharmacological and kinetic properties on the assembled receptors [38]. Thus, receptors containing of GluN1/GluN2B or GluN1/GluN2D subunits have significantly slower offset kinetics as compared to receptors containing GluN2A subunits [38]. These subunits are developmentally regulated such that at birth synaptic NMDA receptors are thought to be GluN1/GluN2B heterodimers whereas at mature synapses GluN2B subunits are largely replaced by GluN2A subunits. We therefore investigated the decay kinetics of NMDA receptor-mediated EPSCs in EGFP-positive granule cells in response to stimulation of the medial perforant path (Fig. 8). Immature granule cells exhibited significantly slower NMDA kinetics (τ_w_ = 156±37 ms, n = 4) compared to mature dentate gyrus granule cells (τ_w_ = 104±31 ms, n = 12, p<0.05, Student's t-Test). Consistent with its slow kinetics, application of the GluN2B selective antagonist ifenprodil (5 µM) [39], [40] reduced EPSC amplitude by 57±11% (n = 3; Fig. 8B). For mature granule cells, while the kinetics of the NMDA receptor mediated EPSC was not reported, Ge et al., 2007 have shown that the EPSC is much less sensitive to ifenprodil [41] consistent with the replacement of GluN2B subunits by GluN2A. While NMDA receptor kinetics differed between EGFP-positive cells and mature granule cells, AMPA receptor kinetics and voltage dependent rectification at the same synapses did not (Fig. 8C).

## Discussion

In rodents and some other mammals, dentate gyrus granule cells are developmentally delayed, i.e. significant maturation occurs in the postnatal period [6], [7], [8], [42]. Moreover, these neurons continue to be produced in large numbers until the first postnatal week [1] and the entire population gradually continues to develop a morphologically and physiologically mature phenotype throughout the first month of life. Electrophysiological recordings from granule cells in immature animals (up to postnatal day 28) have shown a diversity of neuronal maturation states [6], [7]. These range from neurons that show a high input resistance and small dendritic trees to those with low input resistance and a more mature phenotype. These different classes of dentate gyrus granule cells are thought to represent neurons in different stages of development [21]. In the adult, the dentate gyrus retains a neurogenic zone in the subgranular region where neurons continue to be born and integrate into the local circuitry throughout life [4]. Recordings from granule cells in adult animals have found the same range of physiological and morphological properties as described in immature animals [7], [9]. Our finding that high input resistance cells fired small action potentials while low input resistant cells fired more and larger action potentials is consistent with these reports suggesting that newborn neurons in the adult undergo the same developmental program [21].

In the DCX-EGFP mouse, EGFP-expressing dentate granule cells co-expressed DCX, PSA-NCAM and calretinin indicating that they are in the early postmitotic differentiation stage of development [25]. These neurons had high input resistances and relatively depolarized resting membrane potentials, similar to those expected from immature neurons [21]. However, the shunting error introduced by their high input resistance suggests that their resting membrane potential is likely to be more hyperpolarized. In response to depolarizing current injections, the majority of neurons discharged small spikes, as expected in relatively immature neurons, although some did approach a more mature phenotype. However, unlike mature granule cells, none of the EGFP-expressing neurons displayed a slow AHP. The AHP is generated by activation of calcium-dependent potassium currents activated by calcium influx during action potentials [35]. As EGFP-expressing neurons had robust calcium transients in response to action potentials, this lack of AHP is not due to the lack of voltage-dependent calcium channels but suggests that development of the AHP current occurs during late maturation.

In mammalian projection neurons, glutamatergic synapses are almost entirely made onto dendritic spines [43]. In developing neurons, data from cultured neurons suggests that a filopodial protrusion is first extended from the dendritic shaft and new synapses form and stabilize on these filopodia, resulting in the formation of an active dendritic spine [44], [45], [46]. As in mature, spiny granule cells, DCX-EGFP-positive neurons received dual component glutamatergic EPSCs by perforant path stimulation. At synapses on DCX-EGFP cells, the kinetics of the NMDA-receptor EPSC, and its pharmacology indicates that the underlying receptors are most likely GluN1/GluN2B-containing heterodimers, consistent with their early stage of maturation [47], [48]. GluN2B containing NMDA receptors are expressed early in development and are necessary for spine stabilization and maturation [49], [50] and our results are consistent with these being relatively immature synapses.

Based on the electrophysiological properties observed here, the bulk of the EGFP-expressing granule cells in the DCX-EGFP mice appear to be in the early stages of postmitotic differentiation, with few cells exhibiting more mature-like physiology and anatomy. It is possible however to target more mature-like EGFP-expressing granule cells by selecting cells that appear less bright and located further from the SGZ, thus verifying this model for use in future studies requiring the *a priori* identification of either early or late-stage newborn granule cells. These data are consistent with previously published studies investigating neurogenesis in inbred mice and confirm the validity of the Swiss Webster DCX-EGFP mouse for use in future studies investigating the effectors and the role of adult neurogenesis.

## Materials and Methods

### Electrophysiology

All procedures were conducted in accordance with the Australian Code of Practice for the Care and Use of Animals for Scientific Purposes, and were approved by the University of Queensland Animal Ethics Committee. Heterozygous bacterial artificial chromosome (BAC) transgenic mice expressing EGFP under control of the doublecortin (DCX) promoter (GENSAT Project at Rockefeller University, strain: Tg(Dcx-EGFP)BJ224Gsat) were bred and maintained on the Swiss Webster background. Male and female mice aged 1 to 6 months were anaesthetized through ambient inhalation of isoflurane prior to decapitation. Brains were removed rapidly and transferred to ice-cold choline chloride artificial cerebrospinal fluid (aCSF) containing (in mM): 118 C_5_H_14_ClNO, 2.5 KCl, 2.5 CaCl_2_, 1.3 MgCl_2_, 1.2 NaH_2_PO_4_, 10 Glucose and 25 NaHCO, bubbled with carbogen (95% O_2_, 5% CO_2_) to yield pH 7.3–7.4. Horizontal sections, 350 µm thick, containing the dentate gyrus were cut using a Leica VT-1000S Vibratome (Leica Microsystems, Wetzlar, Germany), and transferred to normal aCSF (choline chloride replaced with equimolar NaCl) at 32–34°C. Slices were incubated for one hour prior to experimentation and kept heated throughout the day.

Slices were individually placed into a submerged recording chamber and perfused with carbogen gassed aCSF (equimolar NaCl replacing choline chloride) maintained at 34±2°C (TC-324B, Warner Instruments, LLC, Hamden, Connecticut, USA). Microelectrodes (3–6 MΩ) were pulled from borosilicate glass and filled with an internal recording solution containing (in mM): 135 KMeSO_4_, 5 NaCl, 10 Hepes, 2 Mg_2_-ATP, 0.3 Na_3_-GTP, 0.3 EGTA, 0.1 spermine, 7 phosphocreatine, and 8 biocytin (pH = 7.3, ∼290 mOsmol). For synaptic input recordings KMeSO_4_ was replaced with equimolar CsMeSO_4_. Whole-cell recordings were performed on visually identified EGFP containing dentate gyrus granule cells and EGFP negative dentate gyrus granule cells from primarily the outer edge of the granule cell layer. These cells were identified using an upright microscope (Olympus BX50WI, Olympus Optical, Tokyo, Japan) equipped with a fluorescence attachment. Data were collected using Axograph X software (Axograph Scientific, Sydney, Australia) through an Axon Instruments Multiclamp 700B (Molecular Devices, Sunnyvale, CA, USA) and digitized at 20–50 kHz by an ITC-16 A/D converter (InstruTECH, HEKA, Ludwigshafen/Rhein, Germany). For comparison of deactivation kinetics of the NMDA receptor EPSCs, 5–10 evoked EPSCs were recorded at +40 mV with AMPA receptors blocked by 10 µM NBQX, averaged after which the current decays were fitted to a double exponential equation of the form: 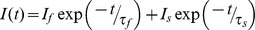 where I_f_ and I_s_ are the amplitudes of the fast and slow and decay components, and τ_f_ and τ_s_ are their respective decay time constants. The weighted time constant was calculated as:
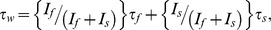
This was used for comparisons of the decay times of the EPSCs.

The data were assumed to follow normal distributions therefore a homoscedastic Student's t-test was used to determine statistical significance between groups. A heteroscedastic t-test was used when the data displayed significantly different variances between groups according to an F-test.

### Immunohistochemistry

Mice were first anesthetized with isoflurane and sodium pentobarbital (150 mg/kg) then transcardially perfused with 2% sodium nitrite (in 0.1 M phosphate buffer (PB), pH 7.4) followed by 50 ml of 4% paraformaldehyde in PB. Brains were removed and postfixed for 2 hours at room temperature then washed in 0.1 M phosphate buffered saline (PBS, pH 7.4) and stored at 4°C overnight. Prior to sub-sectioning, brains were imbedded in 4% agarose (dissolved in H_2_O). Blocks were then transferred back in to PBS and 50 µM thick coronal sections containing the hippocampus were cut using a Vibratome (VT1000S; Leica). All sections were blocked in 5% BSA and 0.05% saponin in PBS for 1 hour then incubated with one of the following primary antibodies (in 0.5% BSA, 0.05% saponin and 0.05% sodium azide in PBS at RT for >60 hours): rabbit polyclonal anti-DCX (1∶2000; Millipore), rabbit polyclonal GFAP (1∶1000; Dako), mouse monoclonal anti-PSA-NCAM (1∶4000; Millipore), rabbit polyclonal anti-calretinin (1∶10,000; Swant), mouse polyclonal anti-CALBINDIN (1∶2000; Sigma-Aldrich), or goat polyclonal anti-NKCC1 (1∶1000; Santa Cruz Biotechnology, Inc.) combined with either rabbit polyclonal anti-GFP (1∶2000; Millipore) or mouse monoclonal anti-GFP (1∶4000; Millipore). For reactions involving a goat primary, sections were then washed in PBS and incubated with a biotinylated donkey anti-goat antibody (1∶500; Jackson ImmunoResearch) in 0.5% BSA, 0.05% saponin and 0.05% sodium azide in PBS at RT for 5 hours. For visualization, sections were washed in PBS and incubated with two of the following fluorescence conjugated secondary antibodies (in 0.5% BSA, 0.05% saponin and 0.05% sodium azide in PBS at RT for 5–16 hours): Alexa Fluor 488 goat anti-mouse (1∶1000; Invitrogen), Alexa Fluor 488 goat anti-rabbit (1∶1000; Invitrogen), Alexa Fluor 555 goat anti-mouse (1∶1000; Invitrogen), Alexa Fluor 555 goat anti-rabbit (1∶1000; Invitrogen), or streptavidin Alexa Fluor 568 (1∶1000; Invitrogen). After additional PBS washes, sections were mounted onto glass slides and coverslips in 50% PBS, 50% glycerol for imaging on an Axio Imager microscope (Zeiss) equipped with an AxioCam MRm/3 camera, ApoTome optical sectioning, Xenon HXP 120 arc lamp, and necessary filter sets for two color fluorescence. ApoTome optical sectioning was used during acquisition of Z-stack images and FIJI software (http://pacific.mpi-cbg.de/wiki/index.php/Fiji) was used to sharpen images, adjust brightness and contrast levels, and compose Z-projection (maximum intensity) and channel merged figures.

### Spine analysis

Slices from electrophysiological recordings with biocytin–filled cells were immersion fixed in 4% paraformaldehyde in 0.1 M phosphate buffer. 50 µm thick sections were cut using a vibratome. Sections were incubated in streptavidin horseradish peroxidase or streptavidin-alexa 555 (1∶2000, Invitrogen). Filled cells were traced using Neurolucida (Microbright field) and total dendritic lengths and spine counts estimated.

### Stereology

Unbiased stereological cell counts were performed using AxioImager Z2 Microscope (Ziess) and images acquired with a 40× objectives an ORCA-R2 digital camera (Hamamatsu). Z stack images were examined using Stereo Investigator 10 (MicroBrightField) to estimate the amount of colocalisation between DCX-GFP positive cells of dentate with other cellular marker including GFAP, DCX, PSA NCAM calbindin, calretinin and NKCC1. Contours were made around the cell body layer of the dentate gyrus and counts were performed using dissectors 15×15×40 µm with a guard zone of 5 µm and a sampling grid of 180×180 µm. (ARC life grant LE100100074)

### BrdU Injections

Mice received one intraperitoneal injection of 25 mg/ml BrdU (5-bromo-2-deoxyuridine: Sigma) in sterile 0.9% NaCl solution (100 mg/kg). 7 and 14 days after the BrdU injections, animals were perfusion fixed with 4% paraformaldehyde and brains removed and post fixed in 4% paraformaldehyde for 2 h. 50 µm thick coronal sections were cut using a vibratome. Sections were pretreated by incubation in 1 M HCl for 20 min at 45°C and washed in 0.1 M Boric acid for 5 min and then 0.1 M PBS 2×5 min. Sections were immunolabeled as described above using mouse monoclonal anti-BrdU (1∶500, Millipore).

*Calcium Imaging*: Two-photon fluorescence images were obtained using an Axioskop 2FS (Zeiss) with a 510 laser scanning head equipped with a Chameleon laser (Coherent) for two photon excitation. For calcium imaging experiments, the green fluorescent calcium indicator Oregon Green BAPTA-1 (OGB-1, 50 µM; Invitrogen) was added to the KMeSO_4_ internal above (minus EGTA and biotin) along with the red fluorescent calcium-insensitive Alexa 594 (30 µM; Invitrogen). OGB-1 and Alexa 594 were excited at 810 nm. The emitted light was split with a dichroic (DT560), bandpass filtered (green channel, 500–560 nm; red channel, 575–640 nm), and detected with separate nondescanned detectors. Fluorescence images were acquired in line scan-mode (100–500 Hz) at a resolution of 10–20 pixels µm^−1^. Apical dendrites were selected at a range of about 20 µm from the soma, or about the first branch point, and the fluorescence over this area was averaged. For two-photon sequences, calcium signals were calculated as the change in green fluorescence (OGB-1) normalized to the red fluorescence (Alexa 594), Δ*G*/*R*. Calcium signals were analyzed off-line. Immediately following the calcium imaging experiments Z-stack images were acquired in the red channel and Z-projection figures were generated off-line using the Zeiss LSM 510 software
